# Impacts of different interactive elements on consumers’ purchase intention in live streaming e-commerce

**DOI:** 10.1371/journal.pone.0315731

**Published:** 2024-12-26

**Authors:** Xiaoli Liu, Lei Zhang

**Affiliations:** 1 College of Economics & Management, Zhejiang University of Water Resources and Electric Power, Hangzhou, People’s Republic of China; 2 Publicity Department, China Jiliang University, Hangzhou, People’s Republic of China; 3 School of Marxism, China University of Mining and Technology, Xuzhou, People’s Republic of China; Sri Sivasubramaniya Nadar College of Engineering, INDIA

## Abstract

Live streaming e-commerce (LSE) has gained tremendous popularity as an innovative social commerce platform that integrates real-time interactions among customers, streamers, and operators to promote product sales. However, there is still much to be discovered about the factors that determine the success of LSE. The objective of this study is to examine the effects of diverse interactive elements, namely consumer-streamer, consumer-platform, and consumer-consumer interactions, on consumers’ purchase intention from the perspectives of social presence and trust using the SOR theory. Additionally, we examine the moderating effects of susceptibility to informative influence on the relationship between different interactive elements and consumers’ purchase intention. We collected survey data from 326 LSE consumers and a structural equation model was employed to evaluate our research hypotheses. Our results reveal that consumer-streamer interaction and consumer-consumer interaction positively influence consumers’ purchase intention. Social presence mediates the relationship between the three types of interactions and consumers’ purchase intention, while trust plays a mediator role in both consumer-streamer and consumer-consumer interactions that affect consumers’ purchase intention. Susceptibility to informative influence has a significant positive moderating effect between consumer-streamer interaction and purchase intention. This study expands on current theoretical research regarding LSE and offers practical insights for operators in the field.

## Introduction

With the development of big data and 5G technology, live streaming has significantly influenced social media users’ usage method and willingness to disseminate information based on the characteristics of interactivity, authenticity, and interconnectedness [1]. Moreover, the extended isolation period triggered by the COVID-19 pandemic has led to the rise of "universal live streaming". Businesses are utilizing live streaming to decrease customer uncertainty and elevate their immersive shopping encounter [2,3]. A novel class of social commerce has surfaced, namely, live streaming e-commerce (LSE), exemplified by Taobao Live. According to CNNIC [4], there existed 777 million live streaming users in China in 2024.

LSE has a significant advantage over traditional social commerce because of its ability to facilitate real-time interactions [5,6]. Interactions in LSE differ from those in social commerce in two significant ways. Firstly, LSE interactions are authentic and visual [7], allowing consumers to gain detailed, dynamic, and real-time product information from streamers who demonstrate product usage and showcase products from various angles [2]. Secondly, LSE interactions happen instantly in real-time [8]; by contrast, in social commerce, if consumers wish to chat with sellers, they must exit the product page [2], without any direct interaction with other consumers. In LSE, consumers can directly engage with streamers and other consumers in real-time via a live chat room. This enables them to ask questions using bullet screens or even start voice conversations through “lianmai.” According to Hu and Chaudhry [7], streamers provide customized product suggestions, advice, and services in a timely manner. Furthermore, consumers can make informed purchase decisions based on other consumers’ experiences shared during live streaming sessions [9]. These interactions can be classified into three forms: consumer-streamer interaction, consumer-platform interaction, and consumer-consumer interaction [10,11]. Consumer-streamer interaction refers to the degree of online interaction between consumers and streamers using communication tools in LSE [12]. Consumer-platform interaction involves consumers’ control over timing, content, and sequence of communication using LSE platforms, as well as the platform’s naturalness and responsiveness [11]. Consumer-consumer interaction refers to consumers’ online interactions with other consumers using communication tools in LSE [12].

Previous research has highlighted the significance of interactivity in LSE. For instance, Ma et al. [1] found that interactivity played a crucial role in shaping consumer responses, while Chen and Liao [6] discovered that it boosted social presence and encouraged viewers to watch live streams. However, most studies have examined interactions as a collective construct, without looking into their individual impacts on consumers. Examining specific interactions systematically in LSE can enhance our understanding of which interactions influence consumers and improve their perceptions. Although several studies have delved into interactions within the context of live streaming environments, their focus has predominantly been on social interactions (e.g., interactions between viewers and streamers, as well as among viewers themselves), with scant attention given to system-level or technical interactions [13,14]. This research diverges from the existing body of work by exploring the interaction between viewers and the platform itself. Gaining a profound comprehension of this viewer-platform dynamic is instrumental in enhancing user interfaces, navigation systems, and latency. Such knowledge can help practitioners optimize communication functions on live streaming platforms to ensure wider interaction spread [11].

E-commerce live streaming has become a crucial field of study, with a range of academic theories being utilized to comprehend consumer behavior and market dynamics. For instance, Affordance Theory [15], Cognitive Transactional Theory [16], Social Exchange Theory [17], Attachment Theory [18], and the Stimulus-Organism-Response (SOR) Theory [19] all contribute to the understanding of the complex interactions within the live streaming e-commerce environment. Hu and Chaudhry [7] emphasize the integral role of interactions in retail environments, proposing their classification as stimuli within the Stimulus-Organism-Response (SOR) paradigm. This approach is supported by the seminal work of Mehrabian and Russell [20], which explores the impact of environmental stimuli on affective and cognitive states, and further developed by Liu et al. [19]. The SOR model is selected for its efficacy in delineating how environmental factors, or stimuli, mold consumer perceptions and emotions, thereby dictating their subsequent behavioral reactions.

In the context of LSE, consumer interactions are considered stimuli, with the resulting purchase intentions constituting the behavioral response. The choice of the SOR model is justified by its ability to capture the dynamic interplay between environmental stimuli (interactions) and consumer responses (purchase intentions), offering a comprehensive lens to analyze the psychological processes that mediate between them. Live streaming enables real-time interactions between viewers and streamers through various communication technologies, providing a unique shopping experience with immersive social presence, reducing uncertainty and risk, and building consumer trust, ultimately influencing viewers’ purchase intention [3,21]. Social presence and trust have been studied as mediating factors between interactivity and outcomes [1,6]; we propose that social presence and trust are likely to be critical mediating factors between interactions and consumers’ purchase intention. The literature on LSE, while growing, has yet to fully explore the multifaceted interactions within the LSE environment. Studies have begun to acknowledge the role of social presence and trust in shaping purchase intentions, yet they often overlook the combined impact of consumer-streamer, consumer-platform, and consumer-consumer interactions [11]. Our study addresses this gap by hypothesizing that these interactions enhance social presence and trust, thereby boosting consumers’ purchase intention. Moreover, the moderating role of susceptibility to informative influence on purchase intention within LSE remains underexplored.

This study investigated how different interactions enhance consumers’ purchase intention using the SOR model. We hypothesize that consumer-streamer, consumer-platform, and consumer-consumer interactions may increase social presence and trust, thereby boosting consumers’ purchase intention. We also investigate the moderating effect of susceptibility to informative influence on consumers’ purchase intention when interacting in LSE. Using survey data collected from 326 Chinese consumers of LSE, we statistically test our hypotheses. This study contributes to live streaming marketing by establishing meaningful links among various interactions, social presence, trust, and consumers’ purchase intention. Our findings can offer valuable insights to e-commerce operators into investment decisions regarding different types of interactions to attract more consumers.

## Theoretical framework and hypothesis formulation

### SOR model

Originating from psychology, the SOR (Stimulus-Organism-Response) model is utilized to elucidate the impact of external environmental stimuli on an individual’s cognitive and emotional condition, which subsequently affects their behavioral responses [20]. A complete SOR model should have stimulus variables, mediating variables, and response variables. Mediating variables play a critical role in expressing the association between environmental stimuli and behavioral responses. The SOR model has found wide-ranging application in the retail domain for comprehending the behavior of online consumers [7,22]. This model is particularly apt for LSE as it allows for the examination of how the interactive nature of live streams can elicit immediate and tangible consumer reactions, aligning with the model’s premise of direct stimulus-response relationships. By employing the SOR framework, we can systematically investigate the psychological underpinnings that link consumer interactions during live streams to their decision-making processes, thereby revealing the nuanced ways in which these interactions can sway purchase intentions. This framework is not only theoretically sound but also practically relevant for understanding and predicting consumer behavior in the evolving landscape of LSE. Therefore, this study suggests that different interactions (S) in LSE (consumer-streamer, consumer-platform, and consumer-consumer interactions) may influence social presence and trust (O), eventually impacting consumers’ purchase intention (R). Therefore, different interactions can relate to social presence and trust and correspondingly enhance consumers’ purchase intention.

### Research framework

The three types of interactions in live streaming e-commerce (LSE) are consumer-streamer interaction, consumer-platform interaction, and consumer-consumer interaction. Consumer-streamer interaction is the direct engagement between consumers and the streamer, leveraging communication tools within LSE to foster a personal connection and influence purchasing decisions. Consumer-platform interaction focuses on the user’s experience with the LSE platform, including control over communication timing, content, and sequence, as well as the platform’s responsiveness and naturalness, which can enhance user satisfaction and engagement. Consumer-consumer interaction involves online communication among consumers, creating a sense of community and shared experience, which can influence social proof and word-of-mouth marketing. In comparison, consumer-streamer interaction has a more direct impact on purchases due to the personal connection, while consumer-platform interaction provides autonomy in the shopping experience, and consumer-consumer interaction contributes to community building and brand loyalty. Each type of interaction plays a unique role in the overall LSE experience, with consumer-streamer interaction being more personal, consumer-platform interaction focusing on user interface and experience, and consumer-consumer interaction fostering a social shopping atmosphere.

**Fig 1:**
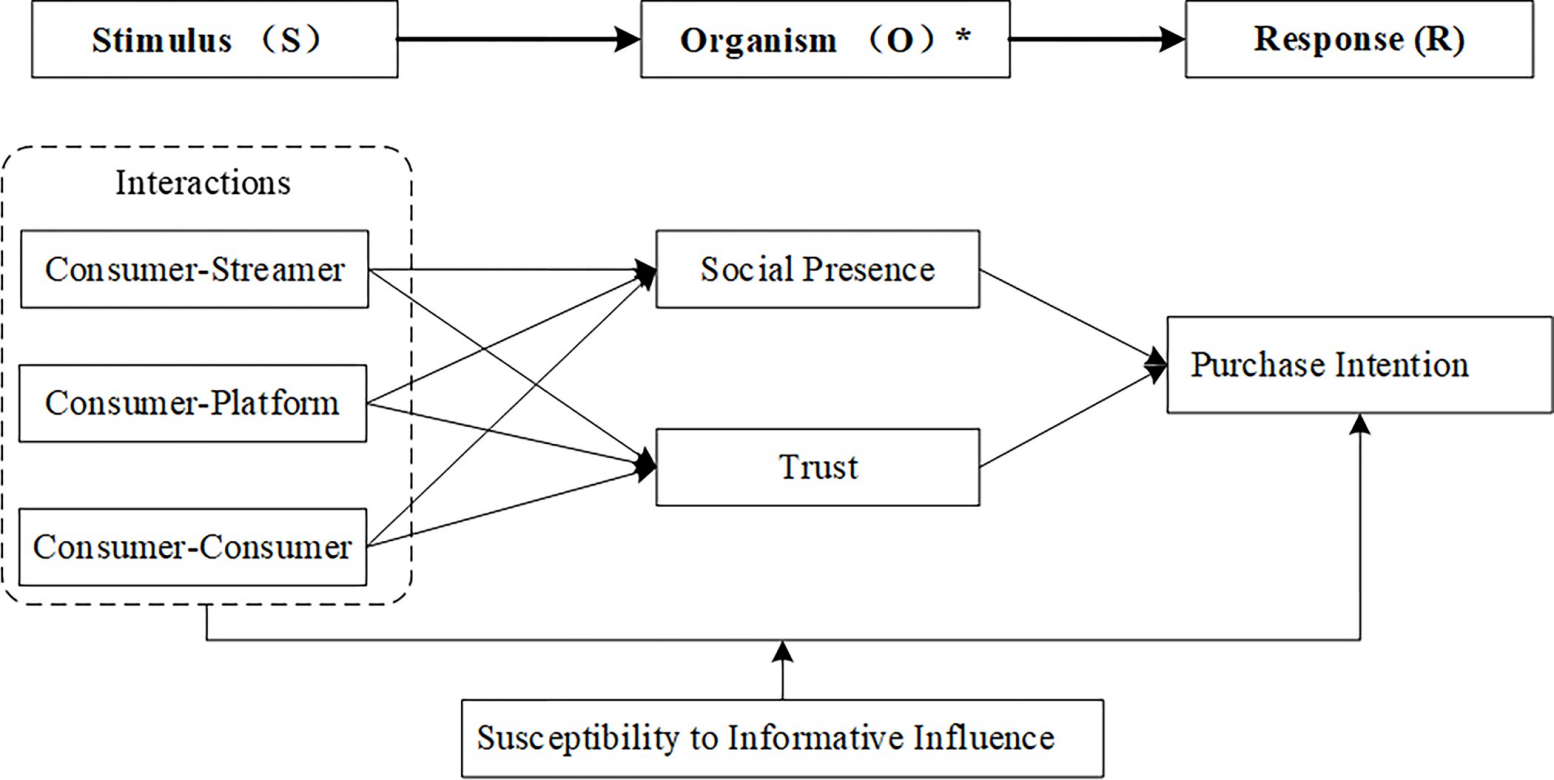
Research model.

The SOR model suggests that various interactions in LSE—including consumer-streamer, consumer-platform, and consumer-consumer interactions—can enhance social presence and trust, leading to an increase in consumers’ purchase intention. Moreover, this study examines the moderating influence of susceptibility to informational influence on the effects of different interactions (i.e., consumer-streamer, consumer-platform, and consumer-consumer interactions) on consumers’ purchase intention in LSE. The research model is illustrated in Fig 1.

### Different interactions and consumers’ purchase intention

Liu et al. [22] discovered a positive correlation between interpersonal interaction factors and flow experience in social commerce, which then impacted purchase intention. Ma et al. [1] argued that when consumers had access to trustworthy information and timely responses to their queries through virtual systems, they were more likely to perceive the information as authentic and useful, leading to an increased likelihood of purchasing online. Chen and Liao [6] demonstrated that interactivity in live streaming positively affected viewers’ willingness to watch streams. Additionally, Ma et al. [1] showed that interactivity played a substantial role in shaping consumer behavioral responses in LSE. Within this context, streamers exhibit merchandise, exchange experiences, and engage with customers using message boards and pop-ups to identify their needs and provide them with real-time information in response to their inquiries [23]. The platforms also use live streaming screens to demonstrate products and interact with consumers through pop-up tweets, purchasing link sharing, and likes during live streaming. Consumers are free to share their purchase and usage experience in the comment section, and through streamers’ responses to other consumers’ questions, they can obtain comprehensive knowledge about the products and brands [24,25]. Consequently, we hypothesize that different interactions in LSE can stimulate consumers’ purchase intention, and propose the following hypotheses:

H1a: Consumer-streamer interactions have a positive impact on consumers’ purchase intention.
H1b: Consumer-platform interactions have a positive influence on consumers’ purchase intention.
H1c: Consumer-consumer interactions have a positive effect on consumers’ purchase intention.

### Different interactions and social presence

Social presence refers to the warm feeling that a medium elicits in the user [26]. It is a psychological perception where the user interacts with others in a virtual space and senses the presence of others. Chen and Liao [6] argued that increased interactivity in live streaming brought viewers closer to the live streaming community. In the two-way interaction scenario of LSE, streamers can showcase products from various angles, exchange product information with consumers, and create an immersive face-to-face shopping experience that potentially gives consumers a sense of social presence and immersion [27–29]. Additionally, factual information presented through the interactions between consumers and the platform allows consumers to see the number of live viewers, creates a warm viewing atmosphere, and provides basic information about the products through the links shared during live streaming [30]. Furthermore, during live streaming, consumers can interact in real-time with each other, and evaluative information conveyed during the live streaming can improve consumers’ knowledge of the products and enhance their shopping experience [31]. As such, we theorize that different interactions in LSE can increase consumers’ social presence. Accordingly, we propose the following hypotheses:

H2a: Consumer-streamer interactions positively impact consumers’ social presence.
H2b: Consumer-platform interactions have a positive effect on consumers’ social presence.
H2c: Consumer-consumer interactions positively influence consumers’ social presence.

### Different interactions and trust

In e-commerce transactions, trust refers to a customer’s willingness to accept uncertainty and purchase from unfamiliar online vendors based on positive expectations [32]. LSE interactions have the potential to fulfil interpersonal needs and alleviate loneliness and psychological distance [33,34]. Feedback from live communication shapes customer psychology and fosters trust in streamers [35–37]. When live streaming enables open access to content, facilitates communication between consumers and streamers and provides timely responses, consumers are more likely to participate, revolve and increase their trust [30]. Greater interaction levels lead to changes that enhance trust [23,24]. Accordingly, we theorize that different interactions in LSE can enhance consumers’ trust. We propose the following hypotheses:

H3a: Consumer-streamer interactions positively influence consumers’ trust.
H3b: Consumer-platform interactions have a positive impact on consumers’ trust.
H3c: Consumer-consumer interactions positively affect consumers’ trust.

### Social presence and consumers’ purchase intention

Previous studies examining social presence have predominantly focused on traditional online shopping platforms [38]. According to Wang et al. [39], enhancing the social presence on B2C websites can be an effective method to promote positive consumer attitudes towards these websites. Sun et al. [40] state that affirmative emotions can solidify the link between consumers and products, motivate customers to take part in product marketing, facilitate the development of a feeling of social presence in the digital realm, and eventually shape purchase intentions. Additionally, Li et al. [41] discovered that social presence in digital social reading acted as a complete mediator between human-to-human interactivity and satisfaction. Social presence helps to bridge the psychological gap between buyers and sellers, fostering a closer relationship between users and sellers or the platform [42–44], improving consumer trust, satisfaction with the product and the live streaming experience [45]. Furthermore, the sense of social presence can be enhanced by sustained and substantial interactions [6], concerns and uncertainties about products can be reduced, and purchase intention can be increased [46]. Therefore, we hypothesize that interactions can impart consumers with a sense of social presence, triggering their purchase intention. Hence, this paper contends for the following hypotheses:

H4: Social presence positively impacts consumers’ purchase intention.
H5a: Social presence mediates the relationship between consumer-streamer interaction and purchase intention.
H5b: Social presence mediates the relationship between consumer-platform interaction and purchase intention.
H5c: Social presence mediates the relationship between consumer-consumer interaction and purchase intention.

### Trust and consumers’ purchase intention

Consumers tend to make quick purchase decisions with limited rationality due to perceived trust [8,47]. Prior research has demonstrated the significance of trust in influencing consumer behavior [19,48]. According to Alkhalifah [48], trust in social commerce has been found to impact behavioral intention. Additionally, Liu et al. [19] showed that trust has a positive influence on purchase intention among consumers of tourism e-commerce live streaming. According to Ma et al. [1], trust serves as a significant factor in reducing uncertainty associated with online shopping, thereby improving consumers’ purchase intentions in LSE. Trust in live-streaming can arise from either the daily interactions between streamers and viewers or the expert skills and abilities of streamers [23]. Moreover, in a study by Liu et al. [49], it was discovered that social support had a direct positive impact on purchase intention in social commerce, with social trust playing a partial mediating role in this association. In tourism e-commerce live streaming, Liu et al. [19] further validated that trust acted as a mediator in the association between the features and consumers’ inclination to make a purchase. Therefore, a hypothesis can be formulated that interactions in LSE creates a sense of trust, leading to an increased willingness among consumers to make purchases. Building upon this, we suggest the following set of hypotheses:

H6: Trust positively impacts consumers’ purchase intention.
H7a: Trust mediates the relationship between consumer-streamer interaction and purchase intention.
H7b: Trust mediates the relationship between consumer-platform interaction and purchase intention.
H7c: Trust mediates the relationship between consumer-consumer interaction and purchase intention.

### The moderating role of susceptibility to informative influence

Susceptibility to informational influence refers to the mechanism through which the attitudes, beliefs, and behaviors of a consumer are impacted by the people in their social surroundings [50–53]. In LSE, consumers obtain useful product information through instantaneous interactions with streamers and other consumers that can facilitate purchase decisions. During this process, consumers’ behaviors may be influenced by the information they receive from others, and their susceptibility to informative influence is closely related to their behavior [54]. According to the interpersonal influence theory, individuals have varying susceptibility to informational influence [53], and the degree to which an individual’s choices and use of products are influenced by others is highly correlated with their level of susceptibility to informational influence [55,56]. Consumers who exhibit a high degree of susceptibility to informational influence tend to rely on the advice or guidance of others to enhance their sense of security [57]. They also perceive others (such as streamers or friends) as credible sources of valuable product information, helping them minimize purchasing risks or losses [54]. Past studies in marketing have provided strong support for the role of susceptibility to informational influence on consumer behaviors [53,58,59]. Therefore, we predict that the effect of different interactions on consumers’ purchase intention will be stronger for those with higher susceptibility to informational influence. Accordingly, we suggest the following set of hypotheses:

H8a: Susceptibility to informational influence moderates the relationship between consumer-streamer interaction and purchase intention in LSE.
H8b: Susceptibility to informational influence moderates the relationship between consumer-platform interaction and purchase intention in LSE.
H8c: Susceptibility to informational influence moderates the relationship between consumer-consumer interaction and purchase intention in LSE.

## Methodology

### Questionnaire design and measurement

In order to guarantee the reliability and validity of our questionnaire, we utilized an established scale and made appropriate adjustments corresponding to LSE characteristics. All constructs were rated on a Likert five-point scale, where 1 denoted "strongly disagree" and 5 symbolized "strongly agree", with higher numbers indicating higher levels of agreement. The measurement of consumer-streamer interaction was primarily based on Cheng [12] and He et al. [11], while items for consumer-platform interaction followed those from He et al. [11]. The consumer-consumer interaction scale was adapted from Long and Tefertiller [60]. Social presence measurement mainly relied on Chen and Liao [6], whereas trust items referred to Wongkitrungrueng and Assarut [3], and susceptibility to informative influence items came from Xue et al. [53]. Finally, the purchase intention scale was adapted from Liu et al. [19]. Our questionnaires underwent review by experts in the field of LSE, and subsequent modifications were made according to their suggestions. We then conducted a pre-survey with 60 participants to improve the initial questionnaire, leading to the formation of the final version used in our study.

### Data collection and sample description

The study was approved by ethics committee of the College of Economics & Management, Zhejiang University of Water Resources and Electric Power. We employed a non-probability sampling technique, specifically a combination of convenience and judgmental sampling, to distribute our questionnaires through the Wenjuanxing app, leveraging WeChat and QQ for wider reach. Participants were pre-screened for prior experience with live streaming e-commerce, ensuring the relevance of our sample [61]. We began by outlining the objective of the study and emphasizing the confidentiality of the responses. Following this, participants provided written consent before proceeding to fill out the questionnaire. In cases where participants were under 18 years old, parental permission was obtained. That is, in the questionnaire, there is a question regarding the participants’ age. If a participant is under 18 years old, they are prompted to have their parent or guardian provide consent. The specific method provided is to require the parent or guardian to electronically sign a consent form, thereby verifying their understanding and approval of their child’s participation. The questionnaire was distributed and gathered responses from January 3 to January 16, 2024. After receiving 381 questionnaires, we excluded incomplete or illogical responses and surveys completed in under one minute, resulting in 326 valid questionnaires with an effective rate of 85.56%.

Table 1 presents the descriptive statistics of our sample. The sample consisted of 326 participants, with 159 males (48.77%) and 167 females (51.23%). The largest age group was comprised of individuals between 25–30 years old, followed by those in the 18–24 years age range. Most respondents held at least a bachelor’s degree, and the largest proportion reported a monthly income of 3,000–5,000 yuan. Over three years of online shopping experience was observed for most participants. Overall, our sample was representative of LSE consumers.

**Table 1:** Descriptive statistics of the study samples. (N = 326).

Variable	Category	Frequency	Percentage (%)
Gender	Male	159	48.77
	Female	167	51.23
Age (years)	Less than 18	16	4.91
	18–24	124	38.04
	25–30	128	39.26
	More than 30	58	17.79
Education level	High school and below	25	7.67
	Junior college	81	24.85
	Bachelor	126	38.65
	Master and above	94	28.83
Income (monthly/yuan)	Under 3,000	96	29.45
	3,000–5,000	135	41.41
	5,000–10,000	52	15.95
	10,000 or more	43	13.19
Online shopping experience (years)	Less than 1	23	7.06
	1–3	76	23.31
	3–5	134	41.10
	More than 5	93	28.53

## Data analysis results

### Reliability analysis

According to Table 2, it is evident that all constructs in our study have a Cronbach’s alpha value above 0.7, demonstrating that our scale possesses high reliability.

**Table 2:** Reliability analysis results.

Constructs	Items	Scales	Cronbach’s alpha
Consumer-streamer interaction (CSI)	CSI1	I interacted with the streamer in the live streaming e-commerce.	0.755
	CSI2	The streamer provided prompt and courteous responses during my interactions.	
	CSI3	The streamer was able to offer me support and advice during my interactions.	
Consumer-platform interaction (CPI)	CPI1	The platform allowed me to have control over my streaming content.	0.785
	CPI2	The interface of the platform was user-friendly, and had good navigation structures.	
	CPI3	The response time for any queries posed by me on the platform was very quick.	
Consumer-consumer interaction (CCI)	CCI1	I interacted with other consumers through chat or private messaging.	0.758
	CCI2	I shared real-time information with other consumers.	
	CCI3	I exchanged streaming content with other consumers.	
Social presence (SP)	SP1	I felt connected and in contact with other consumers while using the platform.	0.805
	SP2	I experienced a sense of socialization whilst using the platform.	
	SP3	I was able to perceive a sense of human warmth while using the platform.	
Trust (TR)	TR1	I believed that the streamer was trustworthy.	0.820
	TR2	The product and service information provided on the live streaming e-commerce platform was considered accurate and truthful.	
	TR3	I had faith that the products I bought would match the ones displayed on the platform.	
Susceptibility to informative influence (SII)	SII1	Prior to making a purchase, I frequently observe what other people are buying and utilizing.	0.941
	SII2	In instances where I lack experience about a product, I frequently seek other’s opinions and experiences.	
	SII3	Before deciding to buy a specific product, I commonly collect information from other users.	
	SII4	When it comes to selecting the best product, I usually consult others for their recommendations on this live streaming e-commerce platform.	
Purchase intention (PI)	PI1	I intend to buy products from this live streaming e-commerce room.	0.754
	PI2	I am likely to purchase products from this live streaming e-commerce room in the future.	
	PI3	For a product I wish to purchase, I will consider buying it from this live streaming e-commerce platform first.	

### Validity analysis

Typically, factor loadings should exceed 0.5, CR values should be above 0.6, and AVE values should exceed 0.5 to demonstrate strong convergent validity [62]. According to the data presented in Table 3, all items have a factor loading greater than 0.6, all CR values exceed 0.7, and the AVE values exceed 0.5. Based on these results, we can conclude that our scale exhibits good convergent validity.

**Table 3:** Convergent validity analysis results.

Constructs	Items	Factor loadings	CR	AVE
Consumer-streamer interaction (CSI)	CSI1	0.773	0.763	0.518
	CSI2	0.704		
	CSI3	0.679		
Consumer-platform interaction (CPI)	CPI1	0.809	0.788	0.554
	CPI2	0.721		
	CPI3	0.698		
Consumer-consumer interaction(CCI)	CCI1	0.757	0.762	0.518
	CCI2	0.630		
	CCI3	0.764		
Social presence(SP)	SP1	0.778	0.805	0.580
	SP2	0.738		
	SP3	0.768		
Trust (TR)	TR1	0.776	0.820	0.603
	TR2	0.744		
	TR3	0.808		
Susceptibility to informative influence (SII)	SII1	0.905	0.941	0.799
	SII2	0.867		
	SII3	0.890		
	SII4	0.912		
Purchase intention (PI)	PI1	0.709	0.753	0.504
	PI2	0.722		
	PI3	0.699		

Table 4 displays the square roots of AVE for each construct along the diagonal. Remarkably, the square root of the AVE value for each construct surpasses the correlation coefficient between that construct and its corresponding constructs [62]. This evidence suggests that our scale has good discriminant validity.

**Table 4:** Discriminant validity analysis results.

**Constructs**	**M**	**SD**	**CSI**	**CPI**	**CCI**	**SP**	**TR**	**SII**	**PI**
CSI	3.708	0.570	0.711						
CPI	3.546	0.630	0.432**	0.718					
CCI	3.480	0.554	0.371**	0.189**	0.736				
SP	3.356	0.693	0.365**	0.283**	0.262**	0.771			
TR	3.623	0.635	0.279**	0.195**	0.214**	0.157**	0.744		
SII	3.582	1.001	-0.014	-0.041	0.146**	0.173**	0.157**		
PI	3.609	0.574	0.447**	0.314**	0.347**	0.363**	0.384**	0.134*	0.715

Note: ** p<0.01

* p<0.05, the numbers in bold on the diagonal are the square roots of the AVE values.

### Common method bias and multicollinearity test

In this study, potential common method bias was addressed through a dual approach, as recommended by Podsakoff et al. [63], by implementing ex-ante measures such as clearly stating the research objectives, ensuring anonymous responses, avoiding semantic ambiguity in question phrasing, and selecting a diverse sample of LSE consumers from different geographic locations. Post-data collection, Harman’s single-factor test was applied, and an exploratory factor analysis with principal component analysis was conducted on all measured items without rotation, revealing that the first principal component explained only 24.967% of the total variance, well below the 50% threshold indicated by Podsakoff et al. [63] and Liu and Zhang [64] for the presence of common method bias, thus concluding that this study is not significantly affected by common method bias.

Variance inflation factor (VIF) is an indicator used to detect multicollinearity. In this study, we assessed the existence of multicollinearity in our model and found that none of the VIF values exceeded 10. This result suggests the absence of multicollinearity problems in our study.

### Hypothesis testing

To test the hypotheses in this study, Structural Equation Modeling (SEM) was utilized. According to the thresholds outlined by Fornell and Larcker [62] and Sun et al. [40], the model fit indices such as χ2/df = 0.350, RMSEA = 0.000, RMR = 0.003, AGFI = 0.992, NFI = 0.999, RFI = 0.985, and CFI = 1.000 indicate that our theoretical model fits the data well.

**Fig 2:**
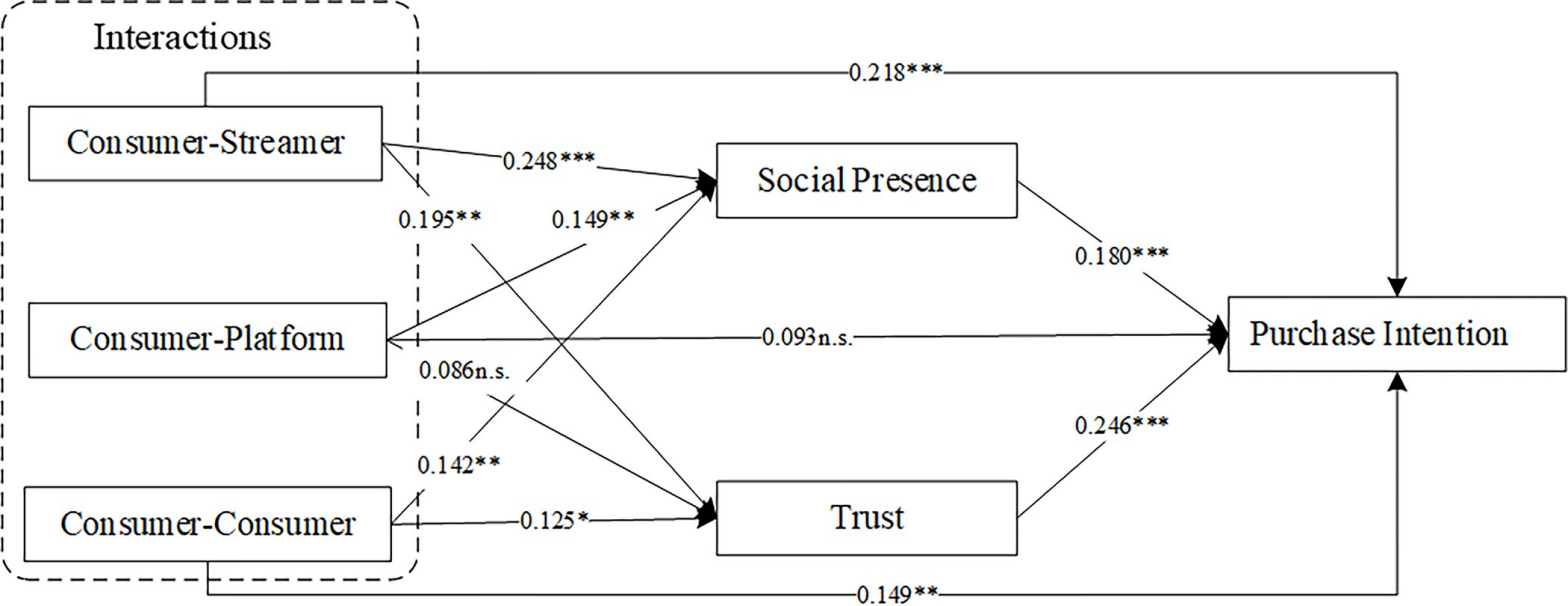
Path coefficient test results. *p < 0.05, **p < 0.01, and ***p < 0.001. n.s., not significant.

The findings in **Table 5** support H1a and H1c, indicating that both consumer-streamer interaction (β = 0.218, p<0.001) and consumer-consumer interaction (β = 0.149, p<0.01) have a positive impact on consumers’ purchase intention. However, the analysis suggests that consumer-platform interaction does not impact consumers’ purchase intention positively (β = 0.093, p>0.05), thereby failing to support H1b. All three interactions—consumer-streamer interaction (β = 0.248, p<0.001), consumer-platform interaction (β = 0.149, p<0.01), and consumer-consumer interaction (β = 0.142, p<0.01)—had a positive effect on social presence, thereby supporting H2a, H2b, and H2c. Both consumer-streamer interaction (β = 0.195, p<0.01) and consumer-consumer interaction (β = 0.125, p<0.05) were shown to have a positive effect on trust, supporting H3a and H3c, but not consumer-platform interaction (β = 0.086, p>0.05), thereby not supporting H3b. Both social presence (β = 0.180, p<0.001) and trust (β = 0.246, p<0.001) positively influenced consumers’ purchase intention, thus supporting H4 and H6. Fig 2 summarizes the findings of the analysis.

**Table 5:** Structural equation model validation results.

Path	Standard path coefficient	Standard error	T value	P value	Hypothesis
CSI→PI	0.218	0.056	3.948	***	H1a: supported
CPI→PI	0.093	0.046	1.828	0.068	H1b: not supported
CCI→PI	0.149	0.051	3.003	0.003	H1c: supported
CSI→SP	0.248	0.072	4.18	***	H2a: supported
CPI→SP	0.149	0.062	2.655	0.008	H2b: supported
CCI→SP	0.142	0.068	2.608	0.009	H2c: supported
CSI→TR	0.195	0.069	3.162	0.002	H3a: supported
CPI→TR	0.086	0.059	1.479	0.139	H3b: not supported
CCI→TR	0.125	0.065	2.199	0.028	H3c: supported
SP→PI	0.180	0.041	3.635	***	H4: supported
TR→PI	0.246	0.043	5.159	***	H6: supported

Note:*** p < 0.001.

To investigate mediating effects, we adopted a bootstrapping procedure with 5000 samples [19]. **Table 6** presents the findings of the analysis. The indirect effect of consumer-streamer interaction on purchase intention, mediated by social presence, was 0.047 (with a 95% confidence interval that does not include 0). Concurrently, the direct effect of consumer-streamer interaction on purchase intention was 0.264 (with a 95% confidence interval that does not include 0). Therefore, social presence exerted a partial mediating effect on purchase intention. Similarly, social presence partially mediated the effects of consumer-platform interaction and consumer-consumer interaction on purchase intention, thereby supporting H5a-H5c. The indirect effect of consumer-streamer interaction on purchase intention, with trust serving as a mediator, was determined to be 0.049 (with a 95% confidence interval that excludes 0). Additionally, the direct effect of consumer-streamer interaction on purchase intention was 0.261 (with a 95% confidence interval that excludes 0). Consequently, trust was found to have a partial mediating effect on purchase intention. Similarly, trust partially mediated the effects of consumer-consumer interaction on purchase intention. However, the indirect effect of consumer-platform interaction on purchase intention via trust as a mediator was only 0.022 (with a 95% CI containing 0). This finding supported H7a and H7c, but not H7b.

**Table 6:** The mediation effects test analysis results.

Path	Estimated	P-value	Bias- corrected 95% confidence interval		Hypothesis
			Lower	Upper	
CSI→PI	0.264	0.001	0.150	0.368	
CSI→SP→PI	0.047	0.000	0.022	0.081	H5a: supported
CPI→PI	0.113	0.041	0.005	0.224	
CPI→SP→PI	0.028	0.007	0.008	0.059	H5b: supported
CCI→PI	0.178	0.000	0.079	0.265	
CCI→SP→PI	0.027	0.002	0.008	0.055	H5c: supported
CSI→PI	0.261	0.000	0.160	0.362	
CSI→TR→PI	0.049	0.002	0.018	0.094	H7a: supported
CPI→PI	0.119	0.020	0.019	0.224	
CPI→TR→PI	0.022	0.119	-0.006	0.057	H7b: not supported
CCI→PI	0.174	0.000	0.078	0.260	
CCI→TR→PI	0.031	0.017	0.006	0.066	H7c: supported

**Fig 3:**
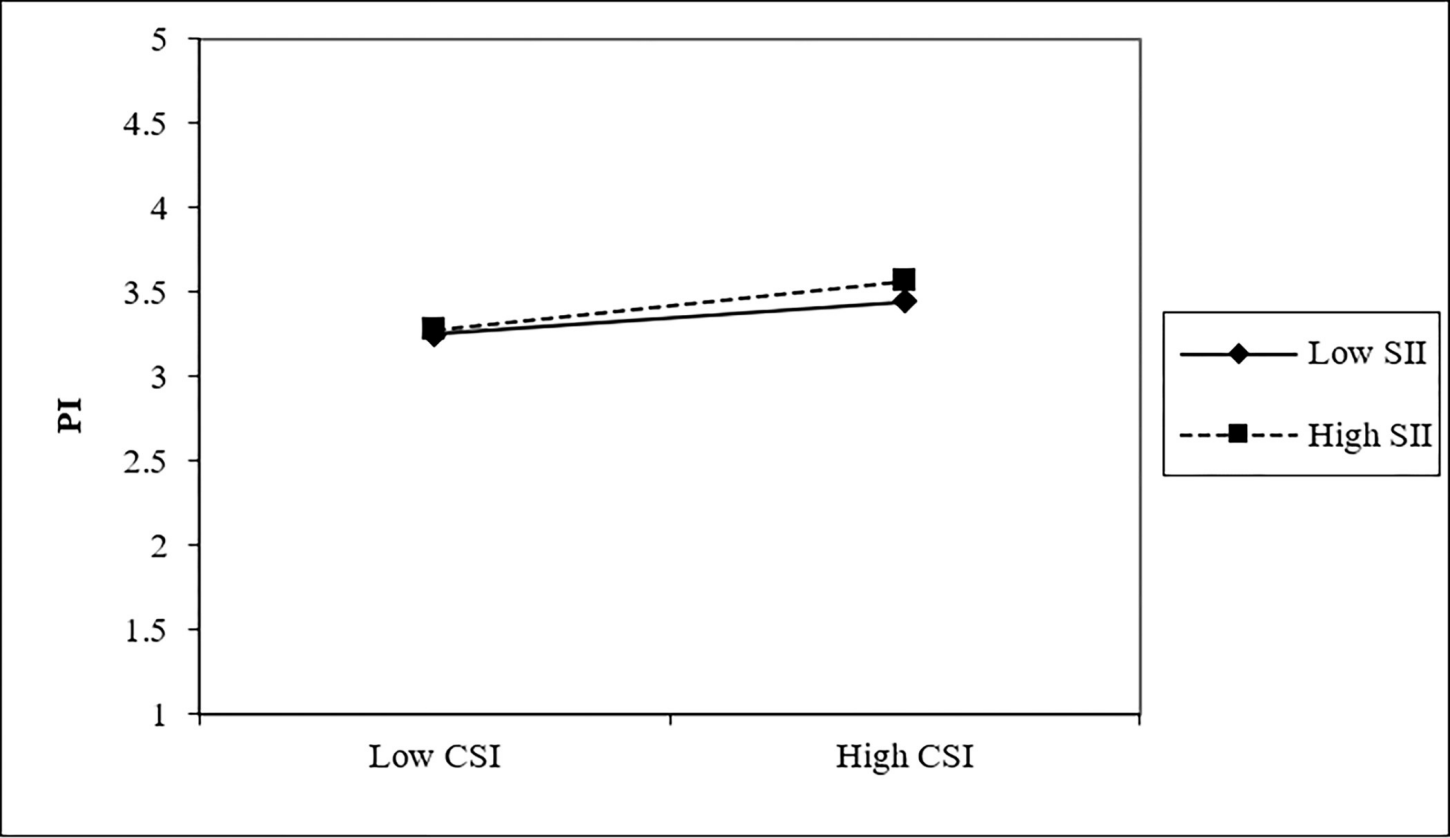
Moderating effect of SII between CSI and PI.

Hayes’ [65] PROCESS macro was employed to investigate the moderating role of susceptibility to informative influence in LSE. The results indicate that there is a significant positive moderating effect of susceptibility to informative influence between consumer-streamer interaction and purchase intention (B = 0.083, t = 2.010, p<0.05, 95% CI [0.002, 0.164]). It means that the impact of consumer-streamer interaction on purchase intention becomes more substantial as susceptibility to informative influence increases (Fig 3), thereby supporting H8a. However, there is no significant moderating effect observed in susceptibility to informative influence for both consumer-platform interaction and purchase intention (B = -0.004, t = -0.077, p>0.05, 95% CI [-0.100, 0.092]) and consumer-consumer interaction and purchase intention (B = 0.038, t = 0.738, p>0.05, 95% CI [-0.063, 0.138]). Consequently, H8b and H8c are not supported.

## Conclusions and implications

### Discussions and conclusions

This study applied the S-O-R model to investigate how different interactions in LSE influence consumers’ purchase intention. We examined the relationships among stimuli (different interactions), organism (social presence and trust), and response (consumer’s purchase intention). Moreover, we also assessed the moderating effect of susceptibility to informative influence on the associations between various interactions and consumers’ purchase intention.

The results of our study indicate that consumer-streamer interaction and consumer-consumer interaction positively influence consumers’ purchase intention. Streamers showcase products, interact with consumers, and address their concerns and doubts by providing them with more real information [23]. Consumers are also free to share their purchase experiences and usage reviews, and the streamers’ responses to other consumers’ questions can assist them in comprehending the products and brands better [24], which leads to an increase in consumers’ willingness to make purchases. There was no significant direct influence of consumer-platform interaction on consumers’ purchase intention, as indicated by our findings. This lack of significance could be attributed to the fact that consumers view interaction with the platform as a fundamental function and are more interested in communicating with streamers and other consumers. Consequently, consumer-platform interaction cannot generate purchase intention directly.

Secondly, our findings indicate that social presence plays a crucial mediating role between various interactions and consumers’ purchase intention. Specifically, we found that consumer-streamer interaction, consumer-platform interaction, and consumer-consumer interaction positively influence social presence, which in turn positively affects consumers’ purchase intention. Streamers can provide consumers with a face-to-face shopping experience by showcasing products from various angles and engaging in information exchange [1,40]. In addition, consumer-platform interaction can create an enthusiastic atmosphere for live viewing by enabling consumers to see the number of live viewers [30], while consumer-consumer interaction can enhance the shopping experience by improving consumers’ product awareness [31]. The continuous and in-depth interactions increase consumers’ sense of social presence [6], evoke positive emotions, lower uncertainties, and alleviate concerns about products, ultimately boosting their willingness to make purchases [41].

Thirdly, trust plays a significant mediating role between consumer-streamer interaction and consumers’ purchase intention, and between consumer-consumer interaction and consumers’ purchase intention. However, we did not find any mediation effect of trust between consumer-platform interaction and consumers’ purchase intention. The findings of our study indicate that interactions between consumers and streamers, as well as interactions between consumers, have a positive impact on trust. This trust then results in an increased purchase intention among consumers. Research conducted by Hou et al. [24] and Zhang et al. [23] demonstrates that when streamers engage with consumers to a significant extent, it can result in cognitive and emotional transformations among consumers. As a result, these consumers have an increased understanding of both the streamer and the products, which ultimately leads to greater levels of trust. Trust plays a critical role as it effectively decreases uncertainty associated with online shopping. Consequently, this reduced uncertainty leads to increased purchase intent among consumers [1]. The non-significant effect of consumer-platform interaction on trust, and the lack of mediation effect of trust between consumer-platform interaction and consumers’ purchase intention, is likely because trust in LSE primarily results from interpersonal interactions, while consumer-platform interaction cannot directly generate trust.

Finally, our study found a significant positive moderating effect of susceptibility to informative influence between consumer-streamer interaction and purchase intention. However, there were no significant moderating effects of susceptibility to informative influence between consumer-platform interaction and purchase intention, or between consumer-consumer interaction and purchase intention. Consumers who have a high susceptibility to informative influence tend to follow others’ guidance and perceive streamers as credible sources of valuable information about the product, reducing their risks or losses when purchasing [54]. Thus, in LSE, the impact of consumer-streamer interaction on purchase intention is stronger for consumers with higher susceptibility to informative influence. Consumer-platform interaction and consumer-consumer interaction do not show a more substantial effect on purchase intention for consumers having lower or higher susceptibility to informative influence.

### Research implications

Firstly, we identified three specific interactions in LSE: consumer-streamer interaction, consumer-platform interaction, and consumer-consumer interaction. In LSE, we studied the effects of these interactions individually on consumers’ purchase intention, thus offering a fresh perspective to quantitative research that typically treats interactions as a whole. While previous studies have shown that interactivity plays a significant role in consumer responses [1] and viewership [6], no previous research has systematically assessed the distinct impacts of specific interactions on consumers. This study extends prior literature by examining the effects of specific interactions on consumers’ perceptions and purchase intentions.

Secondly, our study examined the mediating roles of social presence and trust in linking the three interactions with LSE consumers’ purchase intention, contributing to studies investigating consumers’ purchase intention in this context [66]. In LSE, previous empirical evidence showed that social presence [6] and trust [1] positively affect consumers’ purchase intention. However, they did not integrate the three interactions, social presence, trust, and purchase intention in a unified model. Drawing on the SOR model, our findings reveal that the three interactions indeed relate to social presence, contributing to purchase intention. Furthermore, our results demonstrate that consumer-streamer interaction and consumer-consumer interaction positively influence trust, which contributes to purchase intention. This study presents a novel explanation for the formation of consumer intention in a particular context.

Finally, our research extends the scope of studies on interpersonal influence theory by examining the moderating role of susceptibility to informative influence in LSE, providing new insights into this domain. We found that the effect of consumer-streamer interaction on purchase intention is stronger for consumers with higher susceptibility to informative influence in LSE. Additionally, our results show that consumer-platform interaction and consumer-consumer interaction do not exert a more substantial effect on purchase intention for consumers with lower or higher susceptibility to informative influence, extending the study by Chen et al. [67]. Our research deepens understanding of the boundary conditions and provides insight into the moderating effect of susceptibility to informational influence in LSE.

### Practical implications

The positive effects of different interactions on consumers’ perceptions highlight the need for operators to invest resources in enhancing these interactions during the design of live streams. Streamers should fully utilize the communication tools on the platform, welcome new consumers to the live streaming room, enliven the atmosphere, and address their questions and needs promptly to maximize consumer-streamer interaction. To enhance consumer-platform interaction, platform developers should aid consumers in accessing valuable information about product details through features such as zoomed-in/out pictures and adjusting the size of the live streaming window [53]. LSE operators should optimize functions to establish diverse interaction channels that facilitate real-time communication on product, brand information, and shopping experience among consumers, creating a favorable environment for information exchange and maximizing consumer-consumer interaction.

Social presence mediates between different interactions and consumers’ purchase intention, while trust mediates between consumer-streamer interaction and consumers’ purchase intention, as well as between consumer-consumer interaction and consumers’ purchase intention. Therefore, operators can increase consumers’ purchase intention by enhancing their social presence and trust. This can be achieved by streamers strengthening their professional ability and service quality, communicating with consumers in real-time through timely and effective feedback, and engaging consumers through sweepstakes and fun topics. Streamers can create a relaxed and active environment in the live streaming room, establish an incentive mechanism to encourage consumers to share useful and valuable product information and suggestions in real time, and pay attention to consumers’ behavior (likes, follows, comments, purchases, etc.) during the live stream to provide sincere services that enhance consumers’ dependence and reduce uncertainty. By doing so, streamers can build trust with consumers, making them feel that both the streamer and recommended products are friendly and reliable [54].

In LSE, susceptibility to informative influence moderates the relationship between consumer-streamer interaction and purchase intention. As such, operators should focus on target consumers with high susceptibility to informative influence and take appropriate steps. For consumers with high susceptibility to informative influence, streamers should provide them with better access to information through various forms of interaction, such as responding to their questions through pop-ups to address doubts and offering red packet rewards and other benefits to encourage sharing of product information, thereby increasing their purchase intention.

### Limitations and further research

The study has some limitations. Firstly, since participants completed the questionnaire based on their recent live streaming experience rather than immediately after watching the stream, there may be deviations from real-world scenarios. Future research can use situational experiments to conduct the questionnaire survey. Secondly, apart from susceptibility to informative influence, other individual traits (such as product involvement) that moderate the relationship between different interactions and purchase intention can be explored. Finally, as cultural backgrounds may influence how individuals’ approach and respond to different interactions, cross-cultural studies are necessary. Thus, future research can contribute to the field of LSE by investigating how culture influences its impact.

## Supporting information

S1 File (XLSX)
